# Physiological Trade-Offs Along a Fast-Slow Lifestyle Continuum in Fishes: What Do They Tell Us about Resistance and Resilience to Hypoxia?

**DOI:** 10.1371/journal.pone.0130303

**Published:** 2015-06-12

**Authors:** Rick J. Stoffels

**Affiliations:** 1 Commonwealth Scientific and Industrial Research Organisation, Land and Water, Murray-Darling Freshwater Research Centre, Wodonga, VIC, Australia; 2 Department of Ecology, Environment and Evolution, La Trobe University, Wodonga, VIC, Australia; James Cook University, AUSTRALIA

## Abstract

It has recently been suggested that general rules of change in ecological communities might be found through the development of functional relationships between species traits and performance. The physiological, behavioural and life-history traits of fishes are often organised along a fast-slow lifestyle continuum (FSLC). With respect to resistance (capacity for population to resist change) and resilience (capacity for population to recover from change) to environmental hypoxia, the literature suggests that traits enhancing resilience may come at the expense of traits promoting resistance to hypoxia; a trade-off may exist. Here I test whether three fishes occupying different positions along the FSLC trade-off resistance and resilience to environmental hypoxia. Static respirometry experiments were used to determine resistance, as measured by critical oxygen tension (P_crit_), and capacity for (RC) and magnitude of metabolic reduction (RM). Swimming respirometry experiments were used to determine aspects of resilience: critical (*U*_crit_) and optimal swimming speed (*U*_opt_), and optimal cost of transport (COT_opt_). Results pertaining to metabolic reduction suggest a resistance gradient across species described by the inequality *Melanotaenia fluviatilis* (fast lifestyle) < *Hypseleotris* sp. (intermediate lifestyle) < *Mogurnda adspersa* (slow lifestyle). The U_crit_ and COT_opt_ data suggest a resilience gradient described by the reverse inequality, and so the experiments generally indicate that three fishes occupying different positions on the FSLC trade-off resistance and resilience to hypoxia. However, the scope of inferences that can be drawn from an individual study is narrow, and so steps towards general, trait-based rules of fish community change along environmental gradients are discussed.

## Introduction

Species’ physiological tolerances play an important role in driving spatiotemporal change in abundance, hence community composition [1, 2]. Despite this, biologists that examine the physiology of multiple species, towards understanding the mechanistic basis of community change, have been criticised by ecologists for being too ‘reductionist’ in their approach (discussed in [3]). The validity of this criticism has recently been questioned [4, 5]. Indeed, ‘non-mechanistic’ approaches to community ecology have resulted in a frustratingly poor capacity to explain or predict changes in community composition as a function of environmental change [3, 6, 7]. McGill et al. [4] have argued that greater predictive understanding might be achieved in community ecology through understanding how the physiological performances of key species traits respond to abiotic environmental gradients (the ‘functional-traits approach’). Importantly, the objective is to develop functional relationships between *traits* and performance, not merely between *species* and performance. A functional-traits approach may lead to more general rules of community change, simply because communities around the globe are more likely to share species traits than species *per se* (also see [8]). However, if each individual trait (e.g. standard metabolism) defines an axis in a multi-dimensional trait-space, then it is likely that the set of all traits governing community dynamics would form a very high-dimensional space. A challenge, therefore, is to identify the traits that capture the most information about physiological response to abiotic gradients.

Within numerous animal taxa, ‘lifestyle’ or ‘pace-of-life’ appears to be a trait that is correlated with numerous other physiological, behavioural and life-history traits [9]. With respect to behavioural traits, for example, species with a ‘fast’ lifestyle often have higher rates of dispersal and activity (e.g. movements-per-minute), while species with a ‘slow’ lifestyle are more sedentary [9–11]. Physiological and life-history traits may also be organised along this fast-slow lifestyle continuum (FSLC) [12]. In particular, metabolic rates are correlated with lifestyle, such that fast species have higher metabolic rates than slow species [13–15]. Behavioural, physiological and life-history traits are all likely to have a strong bearing on how species respond to abiotic gradients, and so it follows that lifestyle may be a trait that contains much information about how species will respond to environmental change.

Fish physiologists have, for some time, studied the relationship between lifestyle and physiology. Webb [16] noted the relationship between foraging behaviour, swimming physiology and morphology. Fish with a fast lifestyle may have morphological and physiological traits suiting endurance swimming and active pursuit of prey. Fish with a slow lifestyle have traits suiting burst performance and a sit-and-wait mode of prey capture (also see [17, 18–22]). Trade-offs in physiological performance are evident among fishes along the FSLC [23–28]. For example, the morphological traits that increase endurance performance may reduce burst performance, while the reverse may be true in sedentary fishes [16, 29, 30]. Such performance trade-offs play a very central role in driving pattern and process in ecological communities [31, 32], and so the question arises: how do physiological trade-offs among species along the FSLC shape community response to abiotic gradients?

The objective of the present paper is to examine how physiological trade-offs along the FSLC affect the ‘resistance’ and the ‘resilience’ of fishes to environmental hypoxia (henceforth ‘hypoxia’). The concepts of resistance and resilience have been very useful to ecologists interested in the temporal dynamics of communities subject to disturbance [33, 34–36]. In the present context, a species’ resistance to hypoxia may be viewed as being positively proportional to the magnitude of hypoxia required to drive it ‘locally’ extinct (e.g. extinct within a river reach or channel unit [37]). A species’ resilience to hypoxia is negatively proportional to the time it takes to re-establish a local population, after that local population was driven extinct by hypoxia. Thus resistance and resilience may be viewed, respectively, as the capacity of a population to resist, then recover from, environmental change. This implies that a species can only be resilient to hypoxia if it persists at broader, ‘regional’ scales (e.g. in rivers, the species persists at the scale of the segment or drainage basin [37]).

Environmental hypoxia—along with other physiological stressors—may occur during drought in river networks of Mediterranean climates. Although drought is a natural feature of such systems [38, 39], anthropogenic climate change is likely to increase the frequency and severity of drought in the mid-latitudes [40–45]. Drought presents a particularly interesting problem to aquatic physiologists, for at least two reasons: (1) Drought is likely to cause physiological stress to organisms, such as hypoxia and high temperatures [46, 47]. (2) The science of drought ecology is particularly well suited to a ‘bottom-up’, functional-traits approach (*sensu* [8]), whereby one might aim to experimentally determine effects at the organismal and sub-organismal levels, then scale those effects up to the population and community levels of organisation. The reason for this is that droughts are episodic and their timing is unpredictable, making a field-based scientific understanding of their impacts difficult. A physiological, functional-traits approach to drought ecology will facilitate forecasts of drought-effects on communities, hence management decisions around minimising certain effects, before droughts actually occur.

Several physiological traits contribute to the resistance and resilience of fishes to hypoxia. Traits that increase resistance include: a low critical oxygen tension; plasticity of gill surface area; blood with a high affinity for oxygen; strong capacity for metabolic depression; and high energy reserves to fuel anaerobic metabolism [48–54]. Various aspects of swimming physiology that increase capacity for dispersal may increase resilience, like a low cost of transport [55, 56]. According to the literature, there are good reasons to expect fishes to trade-off resistance and resilience to hypoxia along the FSLC. Fast species generally have high gill surface areas [57] and mitochondrial densities [18] which, respectively, support the high rates of respiration and ATP production required for an active lifestyle and high dispersal capacity [19, 20]. However, while these traits promote the resilience of fast fishes, they may erode their resistance to hypoxia, because high gill surface area and mitochondrial densities both increase metabolic rates [19, 58] which, in turn, may increase critical oxygen tension. Further, Wells [53] has recently hypothesised that fast fishes have several blood physiology traits (low oxygen affinity of haemoglobin; high Hill’s coefficient; large Bohr Effect) that promote the offloading of oxygen to muscle and support an active lifestyle. But these blood physiology traits reduce a fish’s capacity to extract oxygen from the water, and so Wells [53] presents additional reasons to expect a trade-off between dispersal capacity and resistance to hypoxia (but see [52, 59]).

Here I test for a trade-off between resistance and resilience to hypoxia among three fishes with different lifestyles [60]: *Melanotaenia fluviatilis* (Castelnau, 1878; fast lifestyle), *Hypseleotris* sp. (from one population of a broader species complex [61]; intermediate lifestyle) and *Mogurnda adspersa* (Castelnau, 1878; slow lifestyle). These species occupy the same local communities of lowland river-floodplain systems of the Murray-Darling Basin, Australia, where episodic hypoxia may occur as a consequence of droughts [42] or certain flooding events [62]. The three fishes represent a good model system for the question at hand, having lifestyles that span those present in the regional fish fauna. This work builds on that of Dwyer et al. [63], who recently showed that (a) standard and routine metabolic rates (SMR and RMR, respectively) of these three species could be described by the inequality *M*. *fluviatilis* > *Hypseleotris* > *M*. *adspersa*; and (b) their surface respiration behaviour during hypoxia indicated a resistance gradient described by the reverse inequality. It was predicted that a slow lifestyle is associated with high resistance but low resilience to hypoxia, while the reverse is true for fish with a fast lifestyle (low resistance but high resilience). Static respirometry was used to determine critical oxygen tension (P_crit_) and metabolic reduction in each of the three species, where metabolic reduction was estimated as (a) magnitude of reduction (RM) below SMR; and (b) ‘Reduction Capacity’ (RC), a ratio of two areas between curves (A_r_/A_e_), where A_r_ is the area between either SMR (RD_SMR_) or RMR (RD_RMR_) and the observed time series of depressed metabolic rates, during gradual hypoxia, and A_e_ is excess post-hypoxic oxygen consumption (EPHOC [64]). RD_SMR_ is an index of capacity to reduce metabolism below standard rates, and so focuses on physiological down-regulation during hypoxia (*sensu* [49]). In addition to metabolic reduction below standard rates, RD_RMR_ includes any behavioural down-regulation of routine activity as hypoxia ensues, and so adds an alternate view on reduction of aerobic metabolism during hypoxia. Reduction of routine activity—hence RMR—during hypoxia may vary among fishes with different lifestyles [21, 22, 63]. Swimming respirometry was used to determine critical and optimal swimming speed (*U*_crit_ and *U*_opt_, respectively) and cost of transport (COT), both of which contribute to dispersal capacity, hence resilience. Specific hypotheses for each variable are summarised in Table 1.

**Table 1:** Summary of the hypotheses tested (in bold) in this study, using three species of freshwater fish with differing lifestyles: *Mogurnda adspersa*, *Hypseleotris* sp., and *Melanotaenia fluviatilis*.

Species(lifestyle)	Behaviour and habitat	Metabolism	Resistance to hypoxia	Resilience to hypoxia
*M*. *adspersa*(slow)	Benthic sit-and-wait predator	Low SMR and RMR. **Low MMR**. AS (?)	**HighLow P_crit_High RCHigh RM**	**LowLow *U*_crit_Low *U*_opt_High COT_gross_High COT_opt_**
*Hypseleotris*(intermediate)	Benthopelagic	Intermediate SMR and RMR. **Intermediate MMR**. AS (?)	**MediumMedium P_crit_Medium RCMedium RM**	**MediumMedium *U*_crit_Medium *U*_opt_ MediumCOT_gross_ Medium COT_opt_**
*M*. *fluviatilis*(fast)	Pelagic cruising predator	High SMR and RMR. **High MMR**. AS (?)	**LowHigh P_crit_Low RCLow RM**	**HighHigh *U*_crit_High *U*_opt_Low COT_gross_Low COT_opt_**

SMR, RMR, MMR and AS refer to standard, routine and maximum metabolic rate, and aerobic scope respectively. P_crit_ is critical oxygen tension. Two metabolic reduction variables were measured: (1) ‘Reduction Capacity’ (RC), the logarithm of the ratio of two areas (*ln*(A_r_/A_e_)), where A_r_ is the area between either SMR or RMR and the depressed metabolic rate curves, during gradual hypoxia, and A_e_ is excess post-hypoxic oxygen consumption; (2) RM, the magnitude of metabolic reduction as measured by the percentage of SMR depressed during hypoxia. *U*_crit_ and *U*_opt_ are, respectively, the critical and optimal swimming speeds. COT_gross_ and COT_opt_ are the gross and optimal energetic costs of transport, respectively. Values of SMR and RMR for these three species have already been determined by Dwyer et al. [63], which is why they are not hypotheses tested here. MMR may be linked to lifestyle [26], and so it is included in the set of hypotheses towards improving understanding of how lifestyle affects performance along the FSLC. Interspecific variance in AS is a topic of great current debate and so is included in the analysis, but how its magnitude links with lifestyle isn’t clear [26].

## Materials and Methods

This study was carried out in strict accordance with the La Trobe University guidelines for care and use of animals for scientific purposes. All field collection and experimental methods were reviewed and approved under La Trobe University Ethics Permit AEC 11–24 and under VIC Fisheries Permit RP 1014. *M*. *adspersa* are critically endangered and cannot be sourced from the wild, so all individuals were sourced from Narrandera Fish Hatchery (NSW, Australia). All *Hypseleotris* and *M*. *fluviatilis* were sourced from the Broken River (VIC, Australia) using fyke traps, following standard operating procedures reviewed and approved by the La Trobe University Ethics Committee (under AEC 11–24). *Hypseleotris* and *M*. *fluviatilis* were transported to the laboratory in 20 L buckets at low densities (5 per bucket) to minimise stress while in transit (< 1 h). Further details concerning fish collection and accommodation can be found in Dwyer et al. [63] and Allen-Ankins et al. [65]. Water temperature for accommodation and all experiments was fixed at 25°C, a temperature commonly experienced by these fishes in the wild during spring-summer-autumn [60].

### Static respirometry

Computerised, intermittent-flow respirometry was used to estimate metabolic rates of fishes [66]. Each respirometer consists of a glass respiration chamber connected to an oxygen sensor, a flush circuit and a recirculation circuit. Two different chamber sizes were used, depending on the size of the fish: 11 (long) × 2.8 i.d. (internal diameter) cm (70 mL total respirator volume, including tubing) and 9 × 2.2 i.d. cm (38 mL respirator volume). Fibre-optic oxygen sensing was used (PreSens, Regensberg, Germany), and a housing unit ensured fibre cables (bare tip) were held alongside sensor spots within the respiration chambers (Loligo Systems, Tjele, Denmark). Each respirometer was immersed within an ‘ambient tank’ (105 × 47 × 30 cm, maintained at a depth of 17 cm) containing water whose temperature (25°C +/- a range of 0.5°C) and dissolved oxygen (DO) concentrations were monitored and controlled by computers (Loligo Systems, Tjele, Denmark). The measurement circuit of the respirometer enabled recirculation of water during intermittent measurement loops, while the flush circuit enabled flushing of respiration chambers with water from the ambient tank in between measurement loops. Masterflex peristaltic pumps were used for circulation of water through both these circuits, and Masterflex Tygon CHEM tubing was used for its extremely low oxygen permeability (Masterflex, John-Morris Scientific, Chatswood, NSW, Australia). Each measurement loop consisted of three ‘phases’: a ‘flush’, ‘wait’ and ‘measurement’ phase. The flush and measurement phases are self-explanatory; the wait phase enabled stabilisation of the slope of the line describing change of DO within the chamber. The flush, wait and measurement phases for all three species were 180 s / 120 s / 240 s. Blanks were run to control for any background respiration (S1 protocol). The entire respirometry apparatus was situated behind a screen to minimise disturbance from observers.

For estimation of P_crit_ and metabolic depression variables, oxygen consumption rates (${\dot{M}}_{{\text{O}}_{\text{2}}}$, mg O_2_ kg^-1^ h^-1^), were measured over a normoxic, hypoxic and EPHOC period (Excess Post-Hypoxic Oxygen Consumption, following [64]). Fish were placed within chambers at 15:00 on day 1, after which ${\dot{M}}_{{\text{O}}_{\text{2}}}$ was recorded in water with a DO concentration corresponding to 100% air-saturation (20 kPa) over a night-day-night sequence. ${\dot{M}}_{{\text{O}}_{\text{2}}}$ measurements from the first 12 h were excluded as effects of fish stress might have biased estimates of RMR (see below). Following this 12 h acclimation period, ${\dot{M}}_{{\text{O}}_{\text{2}}}$ was collected over a ~ 28 h period to calculate the SMR and RMR of individual *i* (SMR_*i*_ and RMR_*i*_; A minimum of 250 ${\dot{M}}_{{\text{O}}_{\text{2}}}$ measurements were made during the normoxic period). SMR_*i*_ and RMR_*i*_ were calculated as the mean of the lowest 10%, and the mean (respectively) of the ${\dot{M}}_{{\text{O}}_{\text{2}}}$ values from the ~ 28 h normoxic period. Given RMR was calculated over ~ 28 h—hence a day-night sequence—it is unlikely any interspecific differences in diurnal activity patterns biased inferences pertaining to RMR among species. At approximately 09:00 on day 3, hypoxia was induced by bubbling N_2_ gas into an O_2_ stripping column connected to the ambient tank. Oxygen tension was decreased at a rate of 1 kPa every 18 min until 1kPa, after which ambient oxygen tension was maintained at 1 kPa until a fish lost equilibrium, which always occurred just before or shortly after a tension of 1 kPa was reached. A minimum of 40 ${\dot{M}}_{{\text{O}}_{\text{2}}}$ measurements were made during gradual hypoxia. Thus the hypoxic period ended between 14:00 and 17:00 on day 3 of each trial, after which time EPHOC began. The EPHOC period involved returning ambient oxygen tensions to 20 kPa at a rate of ~3.6 kPa h^-1^, and recording ${\dot{M}}_{{\text{O}}_{\text{2}}}$ for a further 24 h after the hypoxic stopping point was reached. A minimum of 100 ${\dot{M}}_{{\text{O}}_{\text{2}}}$ measurements were made during EPHOC. The mean (s.d.) R^2^ values corresponding to individual ${\dot{M}}_{{\text{O}}_{\text{2}}}$ estimates for *M*. *fluviatilis*, *Hypseleotris* and *M*. *adspersa* were as follows (respectively for each species): Normoxic period: 0.98 (0.01), 0.95 (0.03) and 0.97 (0.01); Hypoxic period: 0.98 (0.01), 0.91 (0.02) and 0.87 (0.11); EPHOC: 0.98 (0.05), 0.94 (0.04) and 0.96 (0.02). P_crit_s were estimated for a total of 8 *M*. *fluviatilis*, 9 *Hypseleotris*, and 8 *M*. *adspersa*. Metabolic reduction statistics (RC and RM) were obtained for 6 *M*. *fluviatilis*, 3 *Hypseleotris*, and 3 *M*. *adspersa*. Metabolic reduction statistics were only obtained for individuals that lost equilibrium, and so a lower number of replicates were obtained for RC and RM because not all individuals that yielded P_crit_s lost equilibrium. That is, when four fish are trialled concurrently (the case here), it is most likely that one or two individuals will lose equilibrium before the others, resulting in the ambient tank being re-aerated before the standardised stopping point can be reached for all individuals. Despite the relatively low number of replicates from which RC and RM were estimated, the variances around mean reduction statistics were small, and so tests still had moderate to high power (see Results).

### Swimming respirometry

A Blazka-type, 1.5 L mini-flume was used for swimming energetics experiments [67]. Details of the design can be obtained from www.loligosystems.com (Loligo Systems, Tjele, Denmark). The outer and inner glass tubes had internal diameters of 90 mm and 52 mm, respectively. Two lengths of inner tube were used: 275 mm (*M*. *fluviatilis* and *M*. *adspersa*) and 180 mm (*Hypseleotris*). Black plastic was wrapped around the upper 3^rd^ of the swimming section, which encouraged fish to swim against the current. Water velocity was calibrated against voltage of the motor using dye and a high-speed camera (Optronis, Kehl, Germany). Plastic honeycomb was inserted into both ends of the inner flume to promote micro-turbulent flow. The flume was immersed in a bath whose water was air-saturated, filtered and maintained at 25°C ± a range of 0.2°C. DO tension was measured using a fibre optic dipping probe inserted into the end of the flume (PreSens, Regensberg, Germany). The entire swim flume was situated behind a screen to avoid disturbance from observers. Fish behaviour was monitored using a camera mounted above the flume (uEye, Imaging Development Systems, Germany).

Intermittent-flow respirometry was used to determine ${\dot{M}}_{{\text{O}}_{\text{2}}}$ as a function of water velocity. Individuals used for static respirometry were not reused for swimming respirometry. Fish were placed in the swim flume on the afternoon before each swim trial, and allowed to acclimate for 2 h before a practice swim, after which fish were then allowed to further acclimate to the flume overnight at a velocity of 0.5 body lengths per second (BL s^-1^). During the swim trial, each measurement loop was 8 min in duration (including flush, wait and measurement phases; see Static Respirometry) for all species. Velocity was increased at a rate of 0.5 BL s^-1^ every 16 min, thus enabling two 8 min measurement loops per velocity. Velocity continued to increase at this rate until the fish became exhausted and rested against the downstream honeycomb baffle for longer than 5 s. The breakdown of each measurement loop into flush, wait and measurement phases (see Static Respirometry) was dependent on the size of the fish, and so was species-dependent; phase-times were adjusted to maximise accuracy (R^2^ of ${\dot{M}}_{{\text{O}}_{\text{2}}}$ estimates) without allowing oxygen tension in the chamber to fall below 17 kPa [55]. The flush / wait / measurement times for *M*. *fluviatilis*, *Hypseleotris* and *M*. *adspersa* were, respectively, 100 s / 50 s / 330 s; 0 s / 0 s / 480 s; 100 s / 30 s / 350 s. Due to the small size of *Hypseleotris* relative to the volume of the respirometer, they were swum in a completely closed chamber, but at no stage did oxygen tension fall below 17 kPa. The numbers of swimming trials run for each species were 9 (*M*. *fluviatilis*), 13 (*Hypseleotris*) and 8 (*M*. *adspersa*), but not all trials were retained, due to certain individuals exhibiting unsteady swimming throughout the trial. This resulted in reliable swimming performance data from *N* = 6 (67% success), 7 (54% success) and 6 (75% success) individuals for *M*. *fluviatilis*, *Hypseleotris* and *M*. *adspersa* respectively. The mean (s.d.) R^2^ values corresponding to individual ${\dot{M}}_{{\text{O}}_{\text{2}}}$ estimates for swimming *M*. *fluviatilis*, *Hypseleotris* and *M*. *adspersa* were 0.91 (0.03), 0.82 (0.06) and 0.84 (0.05) respectively. Maximum metabolic rate of individual *i* (MMR_*i*_) was calculated as the maximum ${\dot{M}}_{{\text{O}}_{\text{2}}}$ value obtained during the swimming trial, while aerobic scope of individual *i* (AS_*i*_) was calculated as ${\text{MMR}}_{i}\;-\;\bar{\text{SMR}}$ for each individual, where $\bar{\text{SMR}}$ is the mean SMR of the corresponding species and mass (see Data analysis), obtained using static respirometry. When calculating AS_*i*_, SMR is often calculated from the swimming trial by either assuming some ‘minimum’ metabolic rate in the swim flume is a good approximation of SMR, or by extrapolating the ${\dot{M}}_{{\text{O}}_{\text{2}}}$–velocity function back to a velocity of zero. Neither of these approaches was appropriate in the present case. Although a mini-flume was used for swimming respirometry, individuals still had sufficient space within the flume for routine movements, even at very low velocity, making minimum metabolic rate in the flume an elevated and biased estimate of SMR. Moreover, when SMR estimates were obtained by extrapolation of ${\dot{M}}_{{\text{O}}_{\text{2}}}$ to zero velocity, SMR estimates were noticeably higher than those obtained using static respirometry. Given AS_*i*_ is meant to be an index of the amount of oxygen or energy available to fuel functions additional to SMR, the method used here was deemed more accurate than calculating SMR from swimming trials.

### Data analysis

Oxygen consumption rate, ${\dot{M}}_{{\text{O}}_{2}}$(mg O_2_ kg^-1^ h^-1^), was calculated using:

$${\dot{M}}_{{\text{O}}_{2}}=-(\Delta O_{f}-\Delta O_{b})V_{resp}\alpha B^{-1}$$ (1)

where Δ*O*_*f*_ is the rate of change in oxygen tension (kPa h^-1^) due to fish respiration, Δ*O*_*b*_ is the rate of change in oxygen tension due to background (microbial) respiration (S1 protocol), *V*_*resp*_ is the volume of the respirometer (L; minus the volume of the fish, measured by displacement in a measuring cylinder), *α* is the solubility of oxygen in the water at a known temperature and salinity (mg O_2_ L^-1^ kPa^-1^) and *B* is the mass of the individual (kg). Metabolic rates scale allometrically with mass, and so dividing metabolic rates by mass to give mass-specific rates (${\dot{M}}_{{\text{O}}_{2}}$) does not remove the biasing effects of mass differences among species. We can, however, use the following equation to standardise metabolic rates to a common mass (S2 protocol):

$${\dot{M}}_{{\text{O}}_{2},i,t}={\dot{M}}_{{\text{O}}_{2},i,o}{\left(B_{t}/B_{i}\right)}^{\beta }$$ (2)

where ${\dot{M}}_{{\text{O}}_{2},i,o}$ and ${\dot{M}}_{{\text{O}}_{2},i,t}$ are the observed and standardised mass-specific metabolic rates of individual *i*, respectively, *B*_*i*_ and *B*_*t*_ are the observed and standardised masses (= 3 g here; the mean mass of all individuals used in static respirometry, calculated across species, to the nearest gram), while *β* is the mass-specific allometric scaling exponent [63]. I assumed *β* = -0.247 for all species based on the work of Downs et al. [68]. A fuller discussion of the derivation of Eq 2, as well as the assumptions underlying its use, is presented in the Supporting Information (S2 protocol).

**Fig 1:**
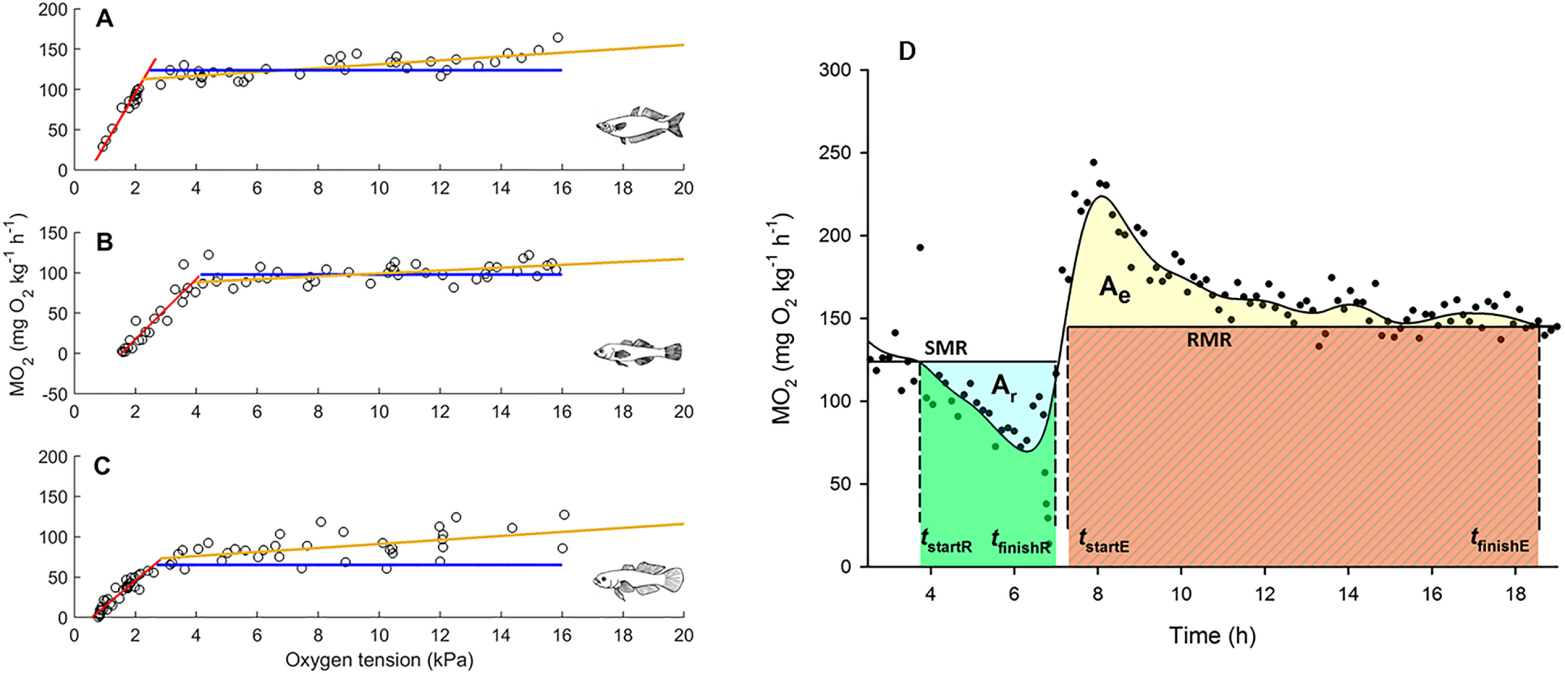
Illustrations of how P_crit_ and metabolic reduction capacity were calculated. A, B and C: Examples of changes in ${\dot{M}}_{{\text{O}}_{2}}$ as a function of oxygen tension for individual *M*. *fluviatilis*, *Hypseleotris* and *M*. *adspersa* (respectively), demonstrating the abrupt decline in ${\dot{M}}_{{\text{O}}_{2}}$ that defines P_crit_. On each plot three lines are presented. The red line is the regression defining the oxyconformation zone of gradual hypoxia, while the orange line is the regression defining the oxyregulation zone during gradual hypoxia. Both the red and orange lines were determined using the algorithm of Yeager and Ultsch, and their point of intersection is one way of calculating P_crit_ (P_crit,YU_). An alternative estimator of P_crit_ (P_crit,SMR_) is the point of intersection of SMR (blue line) and the regression defining oxyconformation (red line). D. Reduction Capacity (RC_SMR_ in this case), was calculated as the logarithm of the ratio of two areas (*ln*(A_r_/A_e_)), where A_r_ is the area between the SMR and depressed metabolic rate curves, during gradual hypoxia, and A_e_ is excess post-hypoxic oxygen consumption (EPHOC). Two points of intersection between the SMR curve and the fitted spline curve, and two points of intersection between the RMR curve and the spline, define four times critical for determination of the integrals defining A_r_ and A_e_: *t*_startR_, *t*_finishR_ *t*_startE_ and *t*_finishE_ are, respectively, the times at which (a) metabolic reduction below SMR began; (b) metabolic reduction ceases; (c) EPHOC begins; (d) EPHOC finishes (see Materials and Methods). (Data presented in D is from a 4.67 g *M*. *fluviatilis*).

P_crit_ values were determined using the algorithm of Yeager and Ultsch [69] (henceforth YU algorithm), which permits an objective, mathematically-explicit, standardised method for determining P_crit_. One could argue, however, that the YU algorithm has one key disadvantage; that P_crit_ is defined as the oxygen tension at which an abrupt change in the slope describing the trend between *mean* ${\dot{M}}_{{\text{O}}_{2}}$ and oxygen tension occurs. Therefore, inasmuch as mean ${\dot{M}}_{{\text{O}}_{2}}$ during gradual hypoxia describes RMR, the YU algorithm may define P_crit_ as the oxygen tension at which RMR can no longer be sustained, rather than the oxygen tension at which SMR can no longer be sustained aerobically. To overcome this potential problem, two estimates of P_crit_ were calculated for each individual: (1) P_crit,YU_, the P_crit_ of Yeager and Ultsch [69]; (2) P_crit,SMR_, determined by the point of intersection between the steep oxyconformation zone and the straight line defined by the SMR of that individual (Fig 1A–1C). For both P_crit,YU_ and P_crit,SMR_ the parameters of the line defining the oxyconformation zone were estimated using the approach described in Yeager and Ultsch [69], hence no subjective ‘eyeballing’ of the data was required. MATLAB code for a modified version of the YU algorithm that returns both P_crit_ estimates is available from the author.

Individual *i*’s magnitude of metabolic reduction during hypoxia, RM_*i*_, was calculated as $[({\text{SMR}}_{i}-M_{{\text{O}}_{2}\text{min},i})\;\cdot \;{\text{SMR}}_{i}^{-1}]\;\cdot \;100$, where $M_{{\text{O}}_{2}\text{min},i}$ is the mean of the three lowest ${\dot{M}}_{{\text{O}}_{2}}$ values obtained during the hypoxic period (invariably at the end of that period, during maximum reduction). Reduction capacity of individual *i*, RC_*i*_, was measured as the logarithm of the ratio of two areas:

$${\text{RC}}_{i}=\text{ln}\left(\frac{{\text{A}}_{\text{r},i}}{{\text{A}}_{\text{e},i}}\right)$$ (3)

where A_r,i_ is the area between the depressed metabolic rate curve and either the SMR (RC_SMR_) or RMR (RC_RMR_) of that individual during gradual hypoxia, and A_e,i_ is EPHOC of that individual following hypoxia. The logarithm is a useful transformation for the ratio of Eq 3 as it centres RC on zero when A_r_ = A_e_, and generates symmetry in the magnitudes of values about zero (e.g. if A_r_ = 2A_e_, ln(A_r_/A_e_) = 0.69, but if 2A_r_ = A_e_, ln(A_r_/A_e_) = -0.69) [70]. Fig 1D is a visual description of how the areas were calculated for RC_SMR_. When estimating the areas for individual *i*, the first step was to fit a spline smoother, *S*_*i*_, to its ${\dot{M}}_{{\text{O}}_{2}}$ time series. The smoothing statistic was fixed at 0.9 for all individuals, yielding mean R^2^ values of 0.83, 0.86 and 0.73, for *M*. *fluviatilis*, *Hypseleotris* and *M*. *adspersa*, respectively. Here, only a description of the equations used to determine RC_SMR_ is provided, but if the reader understands how this index is calculated, then it should be obvious how RC_RMR_ is calculated by making the necessary substitutions of RMR for SMR (as well as associated times, *t*) in Eq 4. Four times are defined by four points of intersection between SMR_*i*_ or RMR_i_ and *S*_*i*_: *t*_startR,i_, *t*_finishR,i_, *t*_startE,i_ and *t*_finishE,i_; the times at which metabolic reduction begins, ends, EPHOC starts and ends, respectively (Fig 1D). The calculations were then straightforward (Fig 1D):

$$\begin{array}{l} {\text{A}}_{\text{r},i}={\text{A}}_{\text{r},\text{SMR},i}-{\text{A}}_{\text{r},\text{spline},i}\\ {\text{A}}_{\text{r,SMR,}i}=(t_{\text{startR},i}-t_{\text{finishR},i})\;\cdot \;{\text{SMR}}_{i}\\ {\text{A}}_{\text{r,spline,}i}=\int_{t_{\text{startR},i}}^{t_{\text{finishR},i}}S_{i}\;dt \end{array}$$ (4)

Similarly (Fig 1D),

$$\begin{array}{l} {\text{A}}_{\text{e},i}={\text{A}}_{\text{e,spline},i}-{\text{A}}_{\text{e,RMR},i}\\ {\text{A}}_{\text{e,spline,}i}=\int_{t_{startE,i}}^{t_{\text{finishE},i}}S_{i}\;dt\\ {\text{A}}_{\text{e,RMR,}i}=(t_{\text{finishE},i}-t_{\text{startE},i})\;\cdot \;{\text{RMR}}_{i} \end{array}$$ (5)

Spline integrals were obtained numerically. We tested for interspecific differences in SMR, RMR, MMR, AS, P_crit,YU_, P_crit,SMR_, RM, RC_SMR_ and RC_RMR_ using one-way ANOVA.

Corrected swimming speeds, *U*_F_, were obtained using:

$$U_{\text{F}}=U_{\text{T}}(1+\varepsilon_{\text{S}})$$ (6)

[71], where *U*_T_ is the velocity in the flume without fish and *ε*_S_ is the fractional error due to solid-blocking (all velocities, *U*, are in units of BL s^-1^). The solid-blocking error is found using:

$$\varepsilon_{\text{S}}=\tau \gamma {\left(A_{\text{O}}\;\cdot \;A_{\text{T}}^{-1}\right)}^{3/2}$$ (7)

where τ is a dimensionless factor depending on flume cross-section shape, and γ is a shape factor for the fish. Here, τ = 0.8 for any sectional shape and γ = 0.5 (body length / body thickness) [71]. Body thickness was calculated as the average of fish depth and width [55]. *A*_O_ is the maximal cross sectional area of the fish (assumed to be an ellipse), and *A*_T_ is the cross sectional area of the flume.

The critical swimming velocity, *U*_crit_, was calculated as

$$U_{\text{crit}}=U_{\text{end-1}}+\frac{t_{\text{end}}}{t_{\text{U}}}U_{\text{end}}$$ (8)

where *U*_end_ is the velocity at which the fish becomes exhausted, *t*_end_ is the time swum at *U*_end_ prior to exhaustion, *t*_U_ is the standard time exposed to a velocity increment (16 min in our case), and *U*_end-1_ is the velocity immediately prior to the one at which exhaustion occurred [72].

Gross COT (Joules km^-1^) was modelled using [73]:

$${\text{COT}}_{\text{gross}}=aU^{-1}+bU^{\text{c-1}}$$ (9)

where *a*, *b* and *c* are parameters. Oxygen consumption rates were converted to Joules by, first, converting to calories using the oxycaloric average of 3.22 cal / mg O_2_ then, second, converting calories to Joules assuming 1 cal = 4.184 J. Some calculus on Eq 9 yields optimum swimming velocity:

$$U_{\text{opt}}={\left(\frac{a}{b(c-1)}\right)}^{1/c}$$ (10)

which, when substituted back into Eq 9 gives the cost of transport at *U*_opt_, COT_opt_ (Joules km^-1^).

In this study the objective was to isolate effects of lifestyle, so all velocities were calculated using the relative units of body lengths per second. If swimming energetics data were analysed using absolute speed (e.g. m s^-1^) then interspecific differences in body length would potentially confound effects of lifestyle. Body size will undoubtedly have an impact on the speed with which fishes swim to recolonise river reaches, hence resilience to disturbance, but for now any effects of body size on resilience to hypoxia were considered beyond the scope of this study.

Non-linear mixed-effects regression was used to model COT_gross_ as a function of *U*; a method appropriate for the repeated measures nature of swim flume experiments [74]. In using this approach each parameter in Eq 9 can be decomposed into a fixed population effect—assumed to be the same each time the population is sampled—and a random effect of the individual fish—sample-dependent random variables. Although the population parameters were of primary interest for modelling COT_gross_, the individual-specific parameter estimates for Eq 9 were used to solve for individual-specific values of *U*_opt_ and COT_opt_ which, in turn, could be analysed using one-way ANOVA. All regression was carried out using MATLAB’s Statistics Toolbox.

## Results

### Metabolic rates and resistance to hypoxia

**Fig 2:**
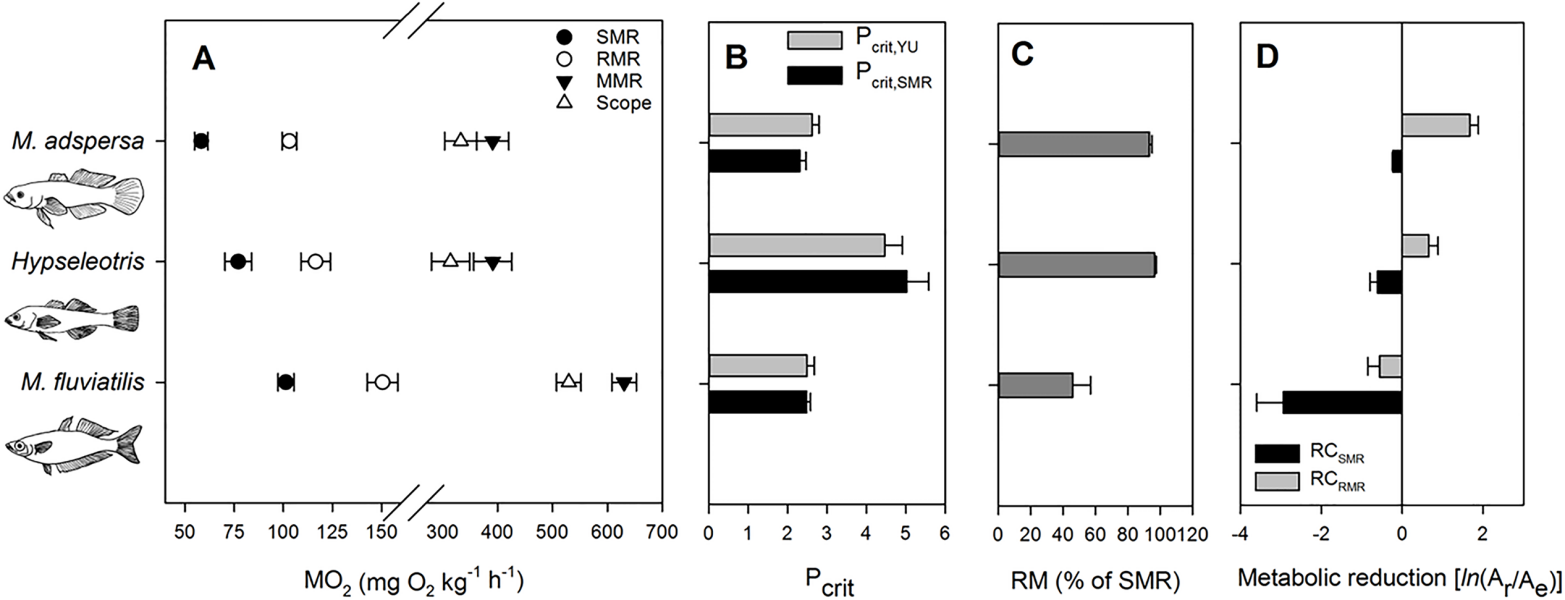
Physiological resistance to hypoxia for three fishes occupying different positions along the fast-slow lifestyle continuum. *Melanotaenia fluviatilis*, *Hypseleotris* sp. and *Mogurnda adspersa* have, respectively, a fast, intermediate and slow lifestyle (Table 1). (A) The gradient in standard (SMR), routine (RMR) and maximum (MMR) metabolic rates, as well as aerobic scope (AS). (B) Critical oxygen tensions (P_crit_s). (C) Magnitude of metabolic reduction. (D) Capacity for metabolic reduction (RC) is the logarithm of the ratio of two areas (*ln*(A_r_/A_e_)), where A_r_ is the area between either SMR (RC_SMR_) or RMR (RC_RMR_) and the depressed metabolic rate curves, during gradual hypoxia, and A_e_ is EPHOC. Means and single standard errors are presented in all plots. Sample sizes for *M*. *fluviatilis*, *Hypseleotris* and *M*. *adspersa* were, respectively: A: N = 16, 16, 12; B: N = 8, 9 and 8; C and D: N = 6, 3 and 3.

The mean masses (s.d.) of *M*. *fluviatilis*, *Hypseleotris* and *M*. *adspersa* used in static respirometry experiments were (respectively) 3.3 g (0.89), 1.4 g (0.79) and 5.4 g (1.19). There were significant interspecific differences in SMR and RMR that were concordant with the data presented by Dwyer et al. [63] (Fig 2A; SMR: F_2_ = 15.86; P < 0.001; Power = 0.99; RMR: F_2_ = 17.39; P < 0.001; Power = 0.99). Although MMR and AS differed significantly among species (Fig 2A; MMR: F_2_ = 20.71; P < 0.001; Power = 1; AS: F_2_ = 15.52; P < 0.001; Power = 0.99), differences in MMR among species were not concordant with the hypothesis; while *M*. *fluviatilis* had a significantly higher MMR and AS than the other two species, MMR and AS did not differ between *Hypseleotris* and *M*. *adspersa* (Fig 2A; Holm-Šídák pairwise comparisons at *α* = 0.05). Both P_crit, YU_ and P_crit,SMR_ yielded very similar estimates of P_crit_ for each species (Fig 2B). P_crit_ values differed significantly among species (P_crit,YU_: F_2_ = 14.13; P < 0.001; Power = 0.99; P_crit.SMR_: F_2_ = 20.46; P < 0.001; Power = 0.1), but not in the pattern expected. Although SMR and RMR could be described by the inequality *M*. *fluviatilis* > *Hypseleotris* > *M*. *adspersa* (Fig 2A), the data collected here did not support the hypothesis that P_crit_ could be described by the same inequality (Fig 2B). The P_crit_ of *Hypseleotris* was higher than those recorded for *M*. *fluviatilis* and *M*. *adspersa*, which did not differ from each other (Fig 2B; Holm-Šídák pairwise comparisons at *α* = 0.05). In contrast with the P_crit_ results, the metabolic reduction results generally supported the hypothesis of a hypoxia resistance gradient described by *M*. *fluviatilis* < *Hypseleotris* < *M*. *adspersa* (Fig 2C and 2D). The magnitude of reduction (RM) varied significantly among fishes (F_2_ = 8.79, P = 0.008; Power = 0.85), with pairwise comparisons indicating significant differences in RM between *M*. *fluviatilis* and both *Hypseleotris* and *M*. *adspersa*, but there was no significant difference in RM between *Hypseleotris* and *M*. *adspersa* (Fig 2C). RC_SMR_ and RC_RMR_ varied significantly and strongly among species (Fig 2D; RC_SMR_: F_2_ = 6.49, P = 0.018; Power = 0.70; RC_RMR_: F_2_ = 14.93, P < 0.001; Power = 0.98), with pairwise tests returning significant differences between *M*. *fluviatilis* and the other two species, but with no significant difference between *M*. *adspersa* and *Hypseleotris* (Fig 2D; only three replicates were available for the pairwise comparison between *Hypseleotris* and *M*. *adspersa* for RC).

### Resilience to hypoxia: dispersal capacity

**Fig 3:**
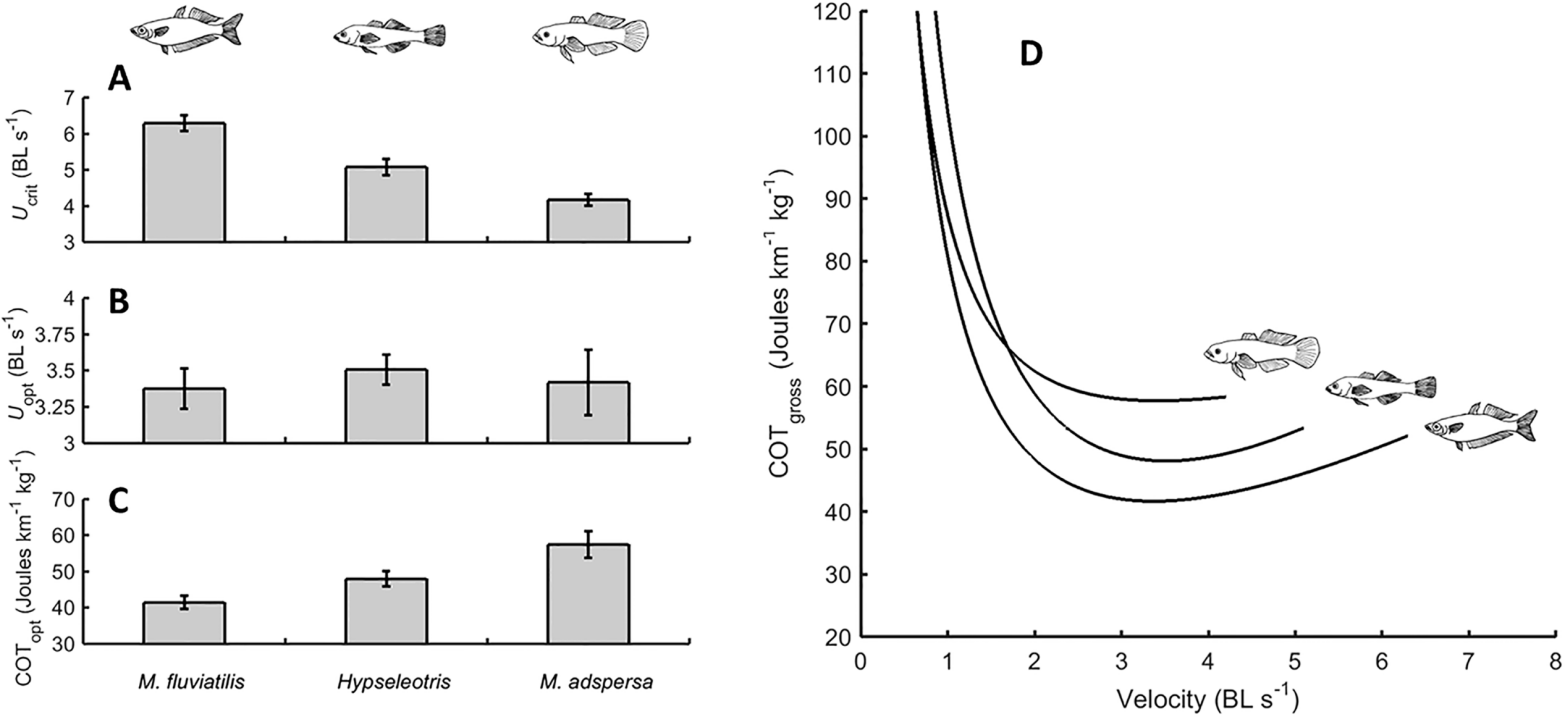
Endurance swimming capacity of three fishes with different lifestyles. *Melanotaenia fluviatilis*, *Hypseleotris* sp. and *Mogurnda adspersa* have, respectively, a fast, intermediate and slow lifestyle (Table 1). A. Critical swim velocities (U_crit_s). B. Mean optimal swimming speed (*U*_opt_) in body lengths per second. (C) Mean gross cost of transport at *U*_opt_. D. Gross cost of transport functions. Lines are modelled relationships between velocity and COT_gross_ using fixed effects estimates of parameters in Eq 9 (estimates in Table 2). Relationships between COT_gross_ and velocity are presented for each species up to their respective mean *U*_crit_ values. All error bars are a single standard error. Sample sizes for *M*. *fluviatilis*, *Hypseleotris* and *M*. *adspersa* were, respectively: N = 6, 7 and 6.

The mean (s.d.) masses and body lengths of *M*. *fluviatilis*, *Hypseleotris* and *M*. *adspersa* used in swimming respirometry experiments were (respectively) 8.2 g (1.1) and 9.2 cm (0.4), 2.8 g (0.89) and 6.0 cm (0.34), and 6.2 g (1.13) and 7.8 cm (0.50). Critical swimming speeds, U_crit_s, could be described by the inequality *M*. *fluviatilis* > *Hypseleotris* > *M*. *adspersa* (Fig 3A), and so support the hypothesis that dispersal capacity is correlated with the FSLC. There were significant differences in the U_crit_ values among species (F_2_ = 25.44, P < 0.001), with all pairwise tests yielding significant differences. Gross COT data also support the hypothesis that adult dispersal capacity is correlated with lifestyle, specifically according to the same inequality described above (Fig 3D; from 2 BL s^-1^ onwards). The parameter estimates and confidence intervals for Eq 9 for each species are given in Table 2. Values of *U*_opt_ were within the range reported for other fishes [73, 75], but there was no significant difference in *U*_opt_ among the fishes studied here (Fig 3B). There was a strong and significant difference in COT_opt_ among fishes (F_2_ = 9.99, P = 0.002), with *M*. *fluviatilis* being the most energetically efficient swimmer, followed by *Hypseleotris* sp. and then *M*. *adspersa* (Fig 3C).

**Table 2:** Key statistics associated with the gross cost of transport function.

Species	*a*	*b*	*c*	*R* ^*2*^
*M*. *fluviatilis*	65.8 ≤ 76.3 ≤ 86.8	2.3 ≤ 4.5 ≤ 6.7	1.9 ≤ 2.2 ≤ 2.5	0.79
*Hypseleotris*	80.6 ≤ 100.9 ≤ 121.2	-1.3 ≤ 3.0 ≤ 7.3	1.6 ≤ 2.5 ≤ 3.4	0.65
*M*. *adspersa*	47.5 ≤ 67.3 ≤ 87.0	6.6 ≤ 20.1 ≤ 33.7	1.1 ≤ 1.5 ≤ 2.0	0.56

Presented are the 95% confidence intervals of the fixed population parameters of Eq 9, as well as the coefficients of determination (R^2^) for Eq 9 fitted to each of the three species tested. Statistics obtained using nonlinear mixed-effects regression to accommodate repeated measures of ${\dot{\text{M}}}_{{\text{O}}_{\text{2}}}$ on individuals across velocities [74]. Sample sizes for *M*. *fluviatilis*, *Hypseleotris* and *M*. *adspersa* were, respectively: N = 6, 7 and 6.

## Discussion

The functional-traits approach to community ecology is currently at the forefront of ecological thinking, and studies that experimentally demonstrate links between organismal traits and performance trade-offs along environmental gradients contribute to the quest for general rules of community change [4]. The results presented here generally support the hypothesis that physiological trade-offs along the FSLC may result in species trading-off resistance and resilience to hypoxia. Coupled with the results of Dwyer et al. [63], the behavioural and metabolic reduction responses to hypoxia suggest a resistance gradient among species described by *M*. *fluviatilis* < *Hypseleotris* < *M*. *adspersa* which, with respect to lifestyle or pace-of-life, equates to a resistance gradient of: fast < intermediate < slow. Inasmuch as dispersal capacity is an indicator of resilience to hypoxia, the *U*_crit_ and COT_opt_ results suggest a resilience gradient—across species or lifestyle—described by the reverse inequality. If this pattern can be generalised (see below), what would a hypoxia resistance-resilience trade-off along the FSLC mean for community response to droughts?

The results of these experiments imply fishes with contrasting lifestyles may exhibit different population-dynamic responses to hypoxia events. As hypoxia ensues within a stream reach, fishes at the fast end of the FSLC may have less capacity to survive hypoxic events due to a low RC. In contrast, slow species may have the physiological traits (high RC) that increase resistance and facilitate local persistence. It follows that, during and immediately after a moderate hypoxic event, the fish community may be dominated by slow species with high resistance.

If, however, hypoxia is sufficiently severe to cause local extinction of the entire fish community (e.g. a drying event), but the community has persisted regionally, fast species with more efficient dispersal capacity may be the first to recolonise a stream reach. For some time horizon after severe hypoxia, the community may then be dominated by fast species. Interestingly, the experimental results reported here agree well with theoretical mechanisms of coexistence in ecological communities, whereby performance trade-offs operating at different spatial scales facilitate regional species coexistence [31].

However, there are several limitations to the scope of inferences that can be made from an individual study like this, and certain performance measures (particularly P_crit_ and *U*_opt_) were discordant with the hypotheses posited in Table 1. The remaining discussion addresses these caveats, before concluding with some statements concerning the importance of furthering a physiological, functional-traits approach to understanding animal resistance-resilience ecology.

Although P_crit_s varied strongly and significantly across fishes, they were clearly uncorrelated with lifestyle. Indeed, the fast, pelagic *M*. *fluviatilis* and the slow, benthic *M*. *adspersa* had almost identical P_crit_s. P_crit_s are generally viewed as being an indicator of resistance to hypoxia [76, 77]. Such an interpretation of the critical oxygen tension assumes that P_crit_ signifies the oxygen tension at which standard metabolism can no longer be maintained aerobically, and any further reduction in oxygen tension results in standard metabolism being increasingly supported by anaerobic pathways, hence the apparent reduction in ${\dot{M}}_{{\text{O}}_{\text{2}}}$ [78]. Under this assumption P_crit_ should be negatively correlated with hypoxia resistance. Alternatively, P_crit_ may indicate the oxygen tension at which an animal depresses its metabolic rate [49]. Under this alternative interpretation, one may view a high P_crit_ as being a trait facilitating resistance to hypoxia, and P_crit_ may be positively correlated with hypoxia resistance. Thus, it is currently not clear exactly what P_crit_ tells us about resistance to hypoxia. One could suggest that RC is a more logical measure of hypoxia resistance, as it explicitly takes into account EPHOC, hence the relative amount of anaerobic metabolism that follows P_crit_ [64]. In the present study, the similar P_crit_s of *M*. *fluviatilis* and *M*. *adspersa* belie their resistance to hypoxia; the RC_RMR_ of *M*. *fluviatilis* was negative, indicating a large A_d_ relative to A_e_ (EPHOC), hence poor capacity for reduction of aerobic metabolism below RMR, while that of *M*. *adspersa* was positive, indicating significantly higher capacity for metabolic reduction below RMR. Further work exploring the ecological relevance of P_crit_ and RC is required.

There is currently much interest in what AS tells us about how species respond to environmental change [26]. In the present study there was no correlation between AS and lifestyle, but one could suggest that a correlation between AS and lifestyle *per se* is not of great value to community ecology. More useful would be an understanding of how lifestyle shapes the partitioning of AS into different functions (e.g. swimming versus digestion) and, in turn, how lifestyle-specific energy budgets then affect resistance and resilience to environmental change. Fu and colleagues have undertaken some seminal work on how lifestyle affects energy budgets [27, 28], but the following step linking different energy budgets to patterns of resistance and resilience is greatly needed.

Certain empirical and theoretical studies have shown that fishes with a morphology conducive to a fast lifestyle should experience less drag, and therefore attain a higher *U*_opt_, than those species with morphological traits associated with a slow lifestyle [30, 75, 79]. In the present study, three species with very different lifestyles all had a similar optimal swimming speed, but the energetic efficiency of swimming at that speed varied across species in the manner hypothesised. It is not uncommon for certain locomotor performance traits like *U*_opt_ to be uncorrelated with morphology or lifestyle [29, 80], and so if one’s objective is to determine the relationship between species traits and performance—in this case steady swimming—then the present study highlights the importance of including multiple measures of performance. Similar to the situation with P_crit_, further work on the relationship between fish lifestyle and *U*_opt_ is required; what is the correlation between *U*_opt_ and COT_opt_ across species with different lifestyles?

The overarching objective of this study was to test whether fishes trade-off resistance and resilience to hypoxia along the FSLC. Like all mechanistic approaches to understanding community dynamics [4], this objective is a very ambitious one. The present study is but one small step towards testing the hypotheses posited in Table 1, and currently the inferences that can be drawn from this study are narrow, for at least four reasons. First, the number of species examined in the manner described here needs to be greatly expanded. Given the amount of work involved in conducting experiments of this nature, this will take patience and a consistent set of protocols across species. Although the three species tested here yield patterns in agreement with the hypotheses of Table 1, at this stage we can have little confidence that the patterns extend to, say, riverine fishes in general.

Second, the present study did not control for the effects of phylogeny. That is, interspecific variation in traits, like lifestyle, may be correlated with phylogeny, and so if we wish to establish robust relationships between traits (not just species) and performance, effects of phylogeny need to be taken into account [81]. To do this in the context of the present problem will require data from more species, as the power to detect phylogenetic signal when examining just three species is extremely low [82]. Until such sample sizes are attained, one could suggest that studies such as the present one and that of Fu et al. [27] encourage important further steps towards achieving a general theory of how physiological trade-offs along the FSLC affect fish community dynamics.

Third, given our ultimate objective is to achieve a trait-based understanding of resistance and resilience to hypoxia, we must remember that the physiological performances examined here are subsets of a broader set of traits that affect resistance and resilience to hypoxia. Capacity for adult dispersal, for example, is only one component of resilience. The passive dispersal of larval stages, as well as the life-history and phenological traits of fishes will also have a bearing on how quickly populations increase following disturbances like hypoxia [83]. Encouragingly, ecologists are beginning to find that the values of numerous behavioural, physiological and life-history traits are correlated across species (the pace-of-life syndrome) [11, 12]. For example, in a recent synthesis Réale et al. [9] hypothesised that fast species, like *M*. *fluviatilis*, may not only have high metabolic rates, but may have behavioural traits favouring high dispersal (e.g. high exploration) [84], and life history traits favouring early reproduction and high growth rates. These behavioural and life history traits will increase population resilience, and so are in general agreement with the hypotheses posited here; that fishes at the fast end of the FSLC will have increased resilience to hypoxia. It seems the prospect for numerous fish resistance-resilience traits to be organised along the FSLC is strong, and numerous exciting hypotheses await testing by experimental biologists.

Fourth, the ultimate test of whether this ‘bottom-up’, functional-traits approach to community ecology is useful, is its ability to explain community dynamics in real ecosystems. It follows that, in addition to overcoming the above three caveats, we then must test whether trait variation along the FSLC explains resistance-resilience dynamics in the wild. Where the data exist, this functional-traits approach to understanding community change along environmental gradients has yielded promising results [85].

The challenges associated with a functional-traits approach to community ecology are daunting, but they are also exciting. One of the reasons the functional-traits approach is exciting is that it encourages a stronger integration of community ecology and physiology. Further, this mechanistic approach involves experimental determination of the performance trade-offs among species with different traits. Given the fundamental importance of trade-offs in shaping the spatiotemporal dynamics of communities [31, 32, 85], experimental physiology has much to offer community ecology. The bridge between community ecology and physiology will be strengthened by focusing on functional relationships between traits and performance, not just species and performance [8].

## Supporting Information

S1 Protocol Correcting for background respiration rates. (PDF)

S2 Protocol Scaling metabolic rates to a common mass. (PDF)
