# Correction: Physiological Trade-Offs Along a Fast-Slow Lifestyle Continuum in Fishes: What Do They Tell Us about Resistance and Resilience to Hypoxia?

**DOI:** 10.1371/journal.pone.0133557

**Published:** 2015-07-15

**Authors:** The PLOS ONE Staff

Table 1 has been corrected for improved readability. The publisher apologizes for the error. Please see the corrected Table 1 here.

**Table 1:** Summary of the hypotheses tested (in bold) in this study, using three species of freshwater fish with differing lifestyles: *Mogurnda adspersa*, *Hypseleotris* sp., and *Melanotaenia fluviatilis*.

Species(lifestyle)	Behaviour and habitat	Metabolism	Resistance to hypoxia	Resilience to hypoxia
*M*. *adspersa*(slow)	Benthic sit-and-wait predator	Low SMR and RMR. **Low MMR.** AS (?)	**High; Low P**_**crit**_ **; High RC; High RM**	**Low; Low *U*crit; Low *U***_**opt**_ **; High COT**_**gross**_ **; High COT**_**opt**_
*Hypseleotris*(intermediate)	Benthopelagic	Intermediate SMR and RMR. **Intermediate MMR.** AS (?)	**Medium; Medium P**_**crit**_ **; Medium RC; Medium RM**	**Medium; Medium *U***_**crit**_ **; Medium *U***_**opt**_ **; MediumCOT**_**gross;**_ **Medium COT**_**opt**_
*M*. *fluviatilis*(fast)	Pelagic cruising predator	High SMR and RMR. **High MMR.** AS (?)	**Low; High P**_**crit**_ **; Low RC; Low RM**	**High; High *U***_**crit**_ **; High *U***_**opt**_ **; Low COT**_**gross**_ **; Low COT**_**opt**_

SMR, RMR, MMR and AS refer to standard, routine and maximum metabolic rate, and aerobic scope respectively. P_crit_ is critical oxygen tension. Two metabolic reduction variables were measured: (1) ‘Reduction Capacity’ (RC), the logarithm of the ratio of two areas (*ln*(A_r_/A_e_)), where A_r_ is the area between either SMR or RMR and the depressed metabolic rate curves, during gradual hypoxia, and A_e_ is excess post-hypoxic oxygen consumption; (2) RM, the magnitude of metabolic reduction as measured by the percentage of SMR depressed during hypoxia. *U*_crit_ and *U*_opt_ are, respectively, the critical and optimal swimming speeds. COT_gross_ and COT_opt_ are the gross and optimal energetic costs of transport, respectively. Values of SMR and RMR for these three species have already been determined by Dwyer et al. [63], which is why they are not hypotheses tested here. MMR may be linked to lifestyle [26], and so it is included in the set of hypotheses towards improving understanding of how lifestyle affects performance along the FSLC. Interspecific variance in AS is a topic of great current debate and so is included in the analysis, but how its magnitude links with lifestyle isn’t clear [26].
