# Supplementary material for: Physiological Trade-Offs Along a Fast-Slow Lifestyle Continuum in Fishes: What Do They Tell Us about Resistance and Resilience to Hypoxia?
Source: PLoS One. 2015 Jun 12;10(6):e0130303. doi: 10.1371/journal.pone.0130303 (PMC4466508; doi:10.1371/journal.pone.0130303)
Supplement: S1 Protocol — (PDF) [file pone.0130303.s001.pdf]

S1 Protocol. Correcting for background respiration rates

In aquatic respirometry, the respiration rate of a fish is estimated as a function of the rate of change in oxygen tension due to the fish (ΔO_f; kPa h^{-1}) within a respiration chamber through time. A potential source of error, therefore, is systematic variation in the rate of change in oxygen tension in the absence of the fish, due to the build-up of microbial communities on the surfaces of the respirometer itself (chamber and tubing; ΔO_b; kPa h^{-1}). One could expect the magnitude of error due to this 'background respiration' to be a positive function of the surface-area-to-volume ratio of the respirometers, given microbes will colonise the surfaces of the respirometer. Accordingly correcting for ΔO_b may be most important when fish biologists are studying fish metabolism using microrespirometry [1].

It follows that oxygen consumption rate, $\dot{V}_{O_2}$(mg O_2 h^{-1}), should be calculated as

$$\dot{V}_{O_2} = -(\Delta O_f - \Delta O_b)V_{resp}\alpha \qquad \text{(S1.1)}$$

where V_{resp} is the volume of the respirometer (L; minus the volume of the fish), and α is the solubility of oxygen in the water at a known temperature and salinity (0.4014 mg O_2 L^{-1} kPa^{-1} in this case). Equation S1.1 is converted to mass-specific respiration rate, $\dot{M}_{O_2}$ (mg O_2 kg^{-1} h^{-1}), by dividing by fish mass, in kg.

ΔO_b was determined in four chambers (2 × 40 ml and 2 × 60 ml chambers; the sizes used in our experiments) situated in the same ambient aquarium used for our fish static respirometry expeiments. Values of ΔO_b were estimated over approximately 68 hours, and exactly the same tubing product (Masterflex Tygon CHEM) and dimensions used for fish experiments were used here (refer to Materials and Methods in the paper). It was not possible to run blanks alongside fish respiration experiments as the measurement phase times [see 2] appropriate for ΔO_f estimates were inappropriate for estimation of ΔO_b.

The rate of growth of a bacterial community colonising a surface might reasonably be described as logistic, or sigmoidal, growth. Because ΔO_b should be negatively proportional to the growth rate of the microbe community, ΔO_b could also be described by a sigmoidal curve, but reflected about the x-axis, hence a negative sigmoidal curve. If this reasoning is true, then an appropriate equation to describe ΔO_b through time (seconds, in this case) is

$$\Delta O_b = \frac{a_{uPart}}{1+\exp(\rho(t-\tau))} + a_l \qquad \text{(S1.2)}$$

where a_l is the lower asymptote in ΔO_b, ι is the time at which the inflection point in ΔO_b occurs, ρ is a slope parameter, and a_{uPart} is part of an equation defining the upper asymptote (a_u) in ΔO_b; $a_u = a_{uPart} + a_l$.

Mean ΔO_b was calculated across the two replicates run for each of the chamber sizes, giving one ΔO_b time series for each chamber size. Non-linear least squares regression was then used to estimate the parameters of Equation S1.2 for each chamber size.

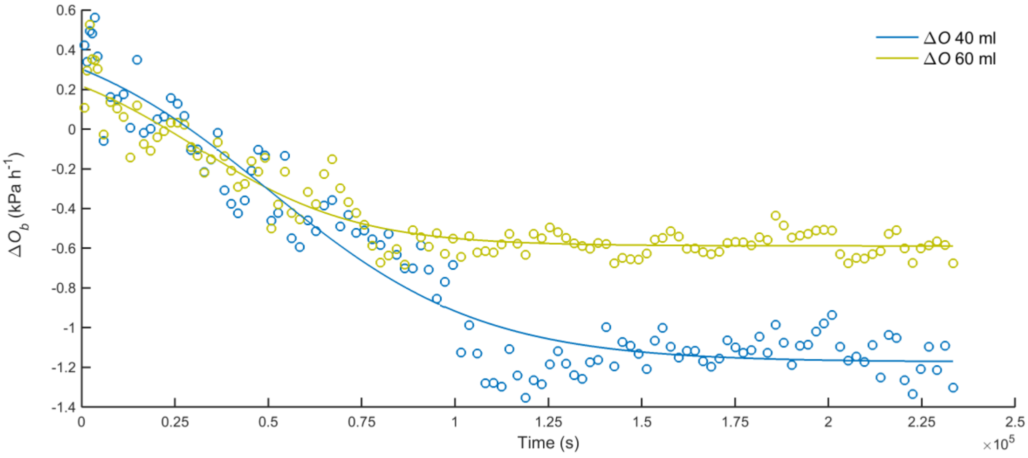

Fig. S1.1. Rates of change in oxygen tension within blank chambers ('background respiration') of two volumes: 40 ml and 60 ml. Rates of change were measured over ~68 hours. Lines defined by a 4-parameter sigmoidal equation (S1.2).

Equation S1.2 described the time series for both chamber volumes very well (Fig. S1.1; Table S1.1). In addition to the high coefficients of determination (Table S1.1), parameter confidence intervals were relatively narrow and were concordant with one might expect. For example, because of the higher surface-area-to-volume ratio of the 40 ml respirometer, one would expect a significantly lower asymptote value, a_l, in this system than in the 60 ml respirometer, which is what was recorded (Table S1.1; Fig. S1.1).

Table S1.1. Statistics associated with the fits of Equation S1.2 to ΔO_b time series from both 40 ml and 60 ml respirometers. Coefficients of determination as well as 95% confidence intervals for each parameter estimate are provided. Parameter definitions are provided in the text.

	R2	a_l	ι	ρ	a_{uPart}
40 ml	0.93	-1.23 ≤ -1.17 ≤ -1.12	38060 ≤ 50290 ≤ 62520	4×10^{-5} ≤ 4.6×10^{-5} ≤ 5.3×10^{-5}	1.39 ≤ 1.73 ≤ 2.06
60 ml	0.90	-0.61 ≤ -0.59 ≤ -0.57	26760 ≤ 30570 ≤ 34380	2.6×10^{-5} ≤ 3.5×10^{-5} ≤ 4.5×10^{-5}	1 ≤ 1 ≤ 1

Background respiration rates generally had a minor effect on estimates of $\dot{M}_{O_2}$ for the three fishes examined. On average, ΔO_b comprised 2.3% (60 ml respirometer), 6.7% (40 ml respirometer) and 2.3% (60 ml respirometer) of the total ΔO. There was a strong left-skew to the frequency distributions of ΔO_b contributions, further indicating ΔO_b was minor relative to ΔO_f (Fig. S1.2).

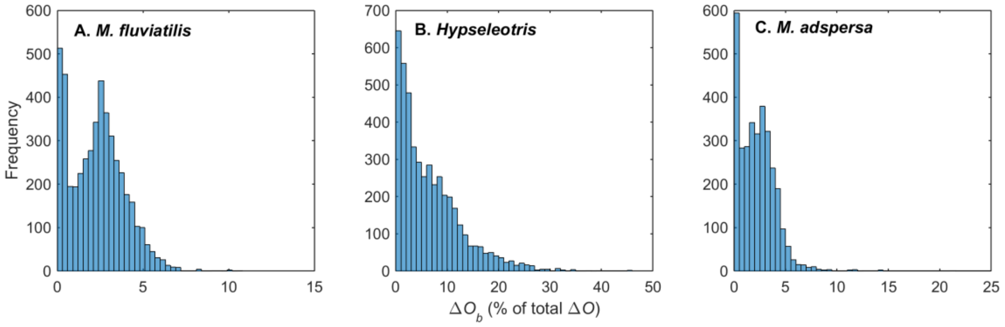

Fig. S1.2. Frequency histograms of ΔO_b expressed as a percentage of total ΔO. Histograms calculated across all individual ΔO estimates for each species.

In Fig. S1.3 time series for both corrected and uncorrected values of ΔO (Fig. S1.3A) and $\dot{M}_{O_2}$ (Fig. S1.3B) are presented for a 1.29 g *Hypseleotris* within the 40 ml respirometer.

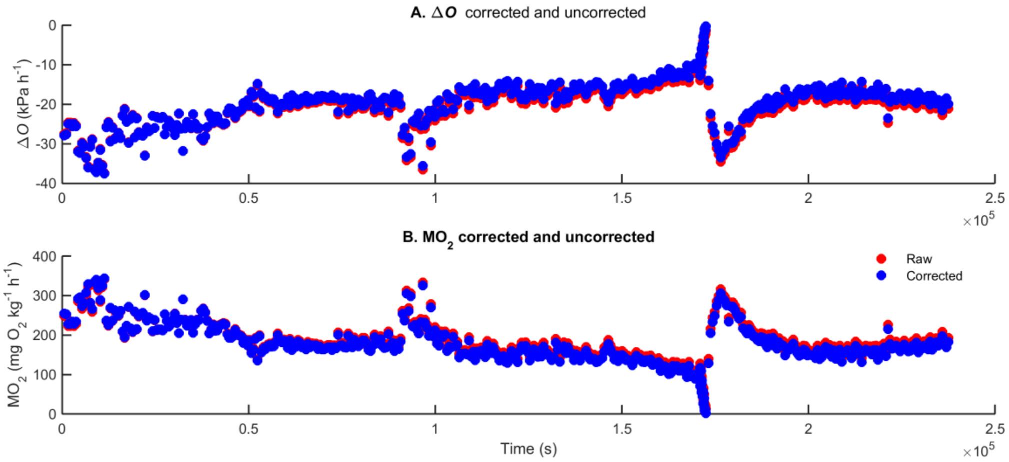

Fig. S1.3. Time series of raw and corrected slopes (ΔO) and $\dot{M}_{O_2}$ estimates for a 1.29 g *Hypseleotris* within a 40 ml chamber. Slopes, hence $\dot{M}_{O_2}$ estimates, corrected using Equation S1.2 and parameter estimates corresponding to a 40 ml chamber in Table S1.1.

S1 References

1. Clark TD, Sandblom E, Jutfelt F (2013) Aerobic scope measurements of fishes in an era of climate change: respirometry, relevance and recommendations. Journal of Experimental Biology 216: 2771-2782.
2. Steffensen JF (1989) Some errors in respirometry of aquatic breathers: How to avoid and correct for them. Fish Physiology and Biochemistry 6: 49-59.
