# Supplementary material for: Physiological Trade-Offs Along a Fast-Slow Lifestyle Continuum in Fishes: What Do They Tell Us about Resistance and Resilience to Hypoxia?
Source: PLoS One. 2015 Jun 12;10(6):e0130303. doi: 10.1371/journal.pone.0130303 (PMC4466508; doi:10.1371/journal.pone.0130303)
Supplement: S2 Protocol — (PDF) [file pone.0130303.s002.pdf]

S2 Protocol. Scaling metabolic rates to a common mass

Metabolic rate scales with body mass according to a power equation [1]. Suppose we obtain a single estimate (subscript *o*; 'observation') of the metabolic rate of individual *i* ($M_{i,o}$) with body mass B_i and error ε_o, then

$$M_{i,o} = \alpha_j B_i^{\beta_j} + \varepsilon_o \qquad \text{(S2.1)}$$

where α_j is the normalisation constant and β_j is the scaling coefficient specific to the *j*th species. With respect to aquatic ectotherms, this observation might be a single rate estimate from an individual measurement loop from an intermittent-flow respirometry system (see Materials and Methods).

While α_j is known to vary significantly across species, β is less variable across taxa, especially within genera, across species with a similar lifestyle and morphology [2-7]. Accordingly, a constant β across all three fish species was assumed, giving a mass-specific scaling exponent of -0.247 (β = 0.753), based on the work of Downs *et al.* [2]. It was also assumed that, while temperature can change the value of α_j, it does not affect the value of β. This also seems a reasonable assumption [3,6]. We do not need to know α_j to scale the metabolic rates of different species to a common mass, as shown below.

Suppose we have obtained an estimate of the metabolic rate of individual *i* with mass B_i at some temperature, but we do not know α_j, nor ε_o. We wish to use this information and equation S2.1 to predict the metabolic rate of this individual at a standard body mass B_t (the mean or modal mass calculated across our study species, say):

$$M_{i,t} = \alpha_j B_t^{\beta} \qquad \text{(S2.2)}$$

We do not need to know α_j, since rearranging S2.1 shows $\alpha_j = (M_{i,o} - \varepsilon_o) B_i^{-\beta}$ which, when substituted into S2.2 gives

$$M_{i,t} = (M_{i,o} - \varepsilon) B_i^{-\beta} B_t^{\beta} \;=\; M_{i,o} \left(\frac{B_t}{B_i}\right)^{\beta} - \varepsilon_o \left(\frac{B_t}{B_i}\right)^{\beta} \qquad \text{(S2.3)}$$

Now suppose we wish to use some set of rescaled individual rate observations to estimate, for example, standard or maximum metabolic rate. To do this we estimate the mean metabolic rate, $\bar{M}$, at B_t, across *n* individual observations:

$$\bar{M}_{i,t} = n^{-1} \sum_{o=1}^{n} M_{i,o} \left(\frac{B_t}{B_i}\right)^{\beta} - n^{-1} \sum_{o=1}^{n} \varepsilon_o \left(\frac{B_t}{B_i}\right)^{\beta} \qquad \text{(S2.4)}$$

If we assume a normal error distribution, then as our sample size of rate estimates increases ($n \to \infty$), the error term of S2.4 vanishes, leaving

$$\bar{M}_{i,t} = \bar{M}_i \left(\frac{B_t}{B_i}\right)^{\beta}$$

Thus, S2.4 enables us to rescale the metabolic rates of all individuals to a common mass, assuming (a) β is invariant across species and temperatures; (b) the errors of individual observations are normally distributed; (c) our particular rate estimates—from standard to maximum—are calculated as means across several individual observations. Equation S2.4

allows for any variation in α_j among species. Note that if S2.4 is used to rescale maximum metabolic rates to a common mass, then we also assume that activity does not significantly affect the mass-specific scaling exponent. Glazier [8] has shown that activity may significantly increase β within species. In the present paper the same value of β was used for rescaling both standard and maximum metabolic rates. It is highly unlikely, however, that this has biased the inferences presented, as Dwyer et al. [9] showed that effects of species-specific variation in β on metabolic rates are minor relative to the large effects of interspecific variance in α_j.

S2 References

1. Schmidt-Nielsen K (1997) Animal Physiology: Adaptation and Environment. Cambridge: Cambridge University Press.
2. Jimenez AG, Locke BR, Kinsey ST (2008) The influence of oxygen and high-energy phosphate diffusion on metabolic scaling in three species of tail-flipping crustaceans. Journal of Experimental Biology 211: 3214-3225.
3. Clarke A, Johnston NM (1999) Scaling of metabolic rate with body mass and temperature in teleost fish. Journal of Animal Ecology 68: 893-905.
4. White CR, Seymour RS (2011) Physiological functions that scale to body mass in fish. In: Farrell AP, editor. Encyclopedia of Fish Physiology: From Genome to Environment: Elsevier Inc. pp. 1573-1582.
5. Savage VM, Gillooly JF, Woodruff WH, West GB, Allen AP, et al. (2004) The predominance of quarter-power scaling in biology. Functional Ecology 18: 257-282.
6. West GB, Brown JH (2005) The origin of allometric scaling laws in biology from genomes to ecosystems: towards a quantitative unifying theory of biological structure and organization. Journal of Experimental Biology 208: 1575-1592.
7. Killen SS, Atkinson D, Glazier DS (2010) The intraspecific scaling of metabolic rate with body mass in fishes depends on lifestyle and temperature. Ecology Letters 13: 184-193.
8. Glazier DS (2009) Activity affects intraspecific body-size scaling of metabolic rate in ectothermic animals. Journal of Comparative Physiology B-Biochemical Systemic and Environmental Physiology 179: 821-828.
9. Dwyer GK, Stoffels RJ, Pridmore PA (2014) Morphology, metabolism and behaviour: responses of three fishes with different lifestyles to acute hypoxia. Freshwater Biology 59: 819-831.
